# Synergistic Effect of Melatonin and Selenium Improves Resistance to Postharvest Gray Mold Disease of Tomato Fruit

**DOI:** 10.3389/fpls.2022.903936

**Published:** 2022-06-22

**Authors:** Huawei Zang, Jiaojiao Ma, Zhilin Wu, Linxi Yuan, Zhi-Qing Lin, Renbin Zhu, Gary S. Bañuelos, Russel J. Reiter, Miao Li, Xuebin Yin

**Affiliations:** ^1^Anhui Province Engineering Laboratory for Green Pesticide Development and Application, Anhui Province Key Laboratory of Crop Integrated Pest Management, School of Plant Protection, Anhui Agriculture University, Hefei, China; ^2^Key Laboratory of Functional Agriculture, Bio-Engineering Research Centre of Selenium, Suzhou Research Institute, University of Science and Technology of China, Suzhou, China; ^3^Anhui Province Key Laboratory of Polar Environment and Global Change, School of Earth and Space Sciences, University of Science and Technology of China, Hefei, China; ^4^Department of Health and Environmental Sciences, Xi’an Jiaotong-Liverpool University, Suzhou, China; ^5^Department of Environmental Sciences, Southern Illinois University Edwardsville, Edwardsville, IL, United States; ^6^Department of Biological Sciences, Southern Illinois University Edwardsville, Edwardsville, IL, United States; ^7^San Joaquin Valley Agricultural Sciences Center, United States Department of Agriculture – Agricultural Research Service, Parlier, CA, United States; ^8^Department of Cell Systems and Anatomy, The University of Texas Health Science Center at San Antonio, San Antonio, TX, United States; ^9^The Central Area of Anhui Province Station for Integrative Agriculture, Research Institute of New Rural Development, Anhui Agricultural University, Hefei, China

**Keywords:** Se-Mel, mineral nutrient, *Botrytis cinerea*, reactive oxygen species, PR proteins

## Abstract

Melatonin (MT) is a ubiquitous hormone molecule that is commonly distributed in nature. MT not only plays an important role in animals and humans but also has extensive functions in plants. Selenium (Se) is an essential micronutrient for animals and humans, and is a beneficial element in higher plants at low concentrations. Postharvest diseases caused by fungal pathogens lead to huge economic losses worldwide. In this study, tomato fruits were treated with an optimal sodium selenite (20 mg/L) and melatonin (10 μmol/L) 2 h and were stored for 7 days at room temperature simulating shelf life, and the synergistic effects of Se and MT collectively called Se-Mel on gray mold decay in tomato fruits by *Botrytis cinerea* was investigated. MT did not have antifungal activity against *B. cinerea in vitro*, while Se significantly inhibited gray mold development caused by *B. cinerea* in tomatoes. However, the interaction of MT and Se showed significant inhibition of the spread and growth of the disease, showing the highest control effect of 74.05%. The combination of MT with Se treatment enhanced the disease resistance of fruits by improving the activities of superoxide dismutase (SOD), peroxidase (POD), and catalase (CAT), as well as increasing the gene expression level of pathogenesis-related (PR) proteins. Altogether, our results indicate that the combination of MT and Se would induce the activation of antioxidant enzymes and increase the expression of PR proteins genes that might directly enhance the resistance in tomato fruit against postharvest pathogenic fungus *B. cinerea*.

## Introduction

The postharvest disease control has become one of the most important limitations in agricultural production in recent years (Tian et al., 2006; Droby et al., 2009; Zhang et al., 2014; Poveda et al., 2020). Among those diseases, postharvest gray mold disease of tomato fruit, which is caused by *Botrytis cinerea*, has been described as one of the most important postharvest diseases of fruit and vegetables worldwide (Qin et al., 2010; Cui et al., 2021). Currently, the research has reported that the application of exogenous chemicals, such as synthetic chemical fungicides, salicylic acid (SA), silicon, either alone or in combination with other treatments as part of the integrated management of postharvest diseases strategies, is also effective in controlling gray mold in harvested tomato fruits (Droby et al., 2009; Soylu et al., 2010; Zheng et al., 2011; Youssef and Roberto, 2014; Wu et al., 2016; Zhu et al., 2016, 2017; Min et al., 2018). However, the growing concern for controlling postharvest diseases with traditional pesticides raises serious concerns about food safety, environmental quality, and pesticide resistance, and the lack of the most effective fungicides have created an interest in exploring for alternative approaches for the disease management (Droby et al., 2009; Wu et al., 2016; Zhu et al., 2016, 2017). In contrast, the integrative plant nutrition is a cost-effective and environmentally friendly way to control the postharvest disease (Wu et al., 2016).

Mineral nutrition has important benefits for plant growth and development as well as has impacts on the plant disease tolerance or resistance, and is recognized as an important component of disease control practices (Datnoff et al., 2007; Cabot et al., 2013; Crane et al., 2014; Brown et al., 2021). The recent information regarding the effect of nutrition on disease tolerance or resistance has been reported by others (Boyd, 2007; Dordas, 2008; Fones et al., 2010; Shi et al., 2011; Yao et al., 2012; Fones and Preston, 2013; Hörger et al., 2013; Yaganza et al., 2014; Vilaplana et al., 2018; Brown et al., 2021). Among those minerals, micronutrient selenium (Se) is an essential trace element for humans and animals at low concentrations, has been found to be beneficial to plants; its effect on the plant–pathogen interactions has little been evaluated, and there are few investigations on Se being used for controlling plant diseases (Hanson et al., 2003; Bañuelos, 2006; Trumble and Sorensen, 2008; Pilon-Smits et al., 2009; Zhao and McGrath, 2009; Zhu et al., 2009, 2016, 2017; Hasanuzzaman et al., 2010; Wu et al., 2014, 2015, 2016; Abdel-Moneim et al., 2022). Therefore, the application of Se at low concentration as possible alternative to synthetic fungicides for the control of plant diseases and producing Se-biofortified agricultural products (Wu et al., 2015, 2016; Schiavon and Pilon-Smits, 2017).

Melatonin (*N*-acetyl-5-methoxytryptamine, MT), a low-molecular-weight organic compound, is a safe and ubiquitous molecule with pleiotropic actions in different organisms, plays pleiotropic roles in plants such as germination and differentiation, ripening and senescence, and acts as a plant biostimulators against various biotic and abiotic stresses such as drought, salt, cold, high temperature, pathogens, herbicides, heavy metals, and ultraviolet radiation (Paredes et al., 2009; Tan et al., 2012; Arnao and Hernandez-Ruiz, 2014, 2015; Reiter et al., 2015; Shi et al., 2015, 2016; Li et al., 2016; Nawaz et al., 2016; Wang et al., 2018; Xu et al., 2019; Yang et al., 2021; Ze et al., 2021; Zhao et al., 2021). Applications of MT in improving postharvest preservation, have been suggested as a useful approach for delaying ripening and senescence processes in plants and improving the storage life and the quality of fruits and vegetables (Sun et al., 2015; Xu et al., 2019; Wang et al., 2020; Zhang et al., 2020; Jayarajan and Sharma, 2021; Di et al., 2022). Thus, one may speculate that the application of MT as potentially important aspect to improve the disease resistance against plant pathogens and produce MT-enriched food crops (cereals, fruits, and vegetables) (Yin et al., 2013; Reiter et al., 2015; Shi et al., 2015; Zhao et al., 2015, 2021; Nawaz et al., 2016; Mandal et al., 2018; Li et al., 2019; Lin et al., 2019; Wang et al., 2019; Ze et al., 2021).

Our previous work has demonstrated that Se could control postharvest disease of fruits and vegetables caused by *Penicillium expansum* and *Botrytis cinerea* (Wu et al., 2014, 2016). However, to our knowledge, little information is available concerning the synergistic effect of MT and Se against postharvest pathogen *B. cinerea* and that induces disease resistance still remains essentially unexplored. Thus, the objective of this study was to evaluate the synergistic effects of combining MT with Se against gray mold in tomato fruit, and the possible mechanisms of disease resistance that is Se-induced and MT-enhanced were also explored by detecting the levels of antioxidant enzymes activities and pathogenesis-related proteins.

## Materials and Methods

### Tomato Fruits and Pathogens

Tomato fruits, Pink 7 variety, were harvested at the commercial maturity stage from Liao Cheng city, Shandong province, China. The fruit without physical damages were surface-sterilized using 2% (v/v) sodium hypochlorite for 2 min, washed with tap water, and air-dried prior to the treatments and analyses.

*Botrytis cinerea* was obtained from the Key Laboratory of Plant Resources in North China, Institute of Botany, Chinese Academy of Sciences. *Botrytis cinerea* was cultivated on the potato dextrose agar (PDA) at 23°C. A mycelium cake with a diameter of 5 mm, from the edge of the growing colony, was inoculated on a 9 cm PDA plate. And after 2 weeks cultivation in incubator at 23°C, spores were scraped from the cultures on the agar surface and diluted into *B. cinerea* conidial suspension of 5 × 10^4^ spores/ml with sterile distilled water containing 0.05% (v/v) Tween 80.

### Treatment

Tomato fruit was wounded with sterile toothpicks at the equator (with a depth of approximately 4 mm), and to each wound was added with 20 μl of the treatment solution that was configured with Se of 20 mg/L and MT of 10 μM, based on the preliminary experiment. Fruit treated with sterile distilled water served as the control. After the wounds were air-dried, fruit was challenge-inoculated with 15 μl of *B. cinerea* at 5 × 10^4^ spores/ml from a conidial suspension. Then, the treated fruits were stored at 25°C with high relative humidity (RH) of about 95%. Gray mold rot was measured 7 days after the treatment. There were three replicates of each treatment, with 20 fruits per replicate. This experiment was repeated twice. The control effect was calculated as follows:


Controleffect(%)=100%×(Control-Treatment)/Control

### Mycelial Growth Assay

To test the effect of Se and MT on the growth of *B. cinerea*, there were three reagent treatments of PDA plate containing different Se and MT concentrations: 20 mg/L Se from sodium selenite (Se), 10 μmol/L MT (MT), and both 20 mg/L Se and 10 μmol/L MT (Se + MT). Petri-dishes with only PDA medium served as the control. Each treatment had four replicates. After inoculation, the petri-dishes were placed in an incubator at 23°C for 48 h, and the colony diameters were measured after a week. The inhibition ratio (%) was calculated as follows:


$$\begin{array}{c} \mathrm{Inhibition}\mathrm{ratio}\left(\%\right)\\ =100\%\times \left(d\mathrm{control}-d\mathrm{treatment}\right)/d\mathrm{control} \end{array}$$

Where “d control” was defined as the diameter of *B. cinerea* mycelium colony with only PDA medium; “d treatment” was defined as the diameter of *B. cinerea* mycelium colony with PDA medium of three reagent treatments (Se, MT, and Se + MT).

### Extraction and Assays of Antioxidant Enzymes and Disease-Related Enzymes

Each fruit sample (0.1 g) was ground in 1% (w/v) polyvinylpolypyrrolidone in a chilled mortar, and then homogenized with 1.2 ml of 50 mM potassium phosphate buffer (pH 7.8) containing 1 mM EDTA-Na2 and 0.3% Triton X-100.

The activity of superoxide dismutase (SOD) was assayed by the inhibition of the photochemical reduction of nitro blue tetrazolium chloride (Agarwal and Pandey, 2004). The catalase (CAT) activity was measured using a spectrometer at 240 nm for the H_2_O_2_ decomposition rate using the extinction coefficient of 40/mM/cm (Havir and McHale, 1987). Peroxidase activity (POD) was determined by monitoring the absorbance increase at 470 nm due to guaiacol oxidation (extinction coefficient of 26.8/mM/cm) (Qin et al., 2010). The glutathione (GSH) concentration was estimated using the spectrophotometry method (Qing et al., 2015), and the polyphenol oxidase (PPO) was determined by the method reported by Soylu et al. (2010).

The guaiacol peroxidase (GPX) activity was measured in the homogenates by the increase in absorption and color change recorded for 5 min at 470 nm due to the guaiacol (hydrogen donor) oxidation (Qing et al., 2015). The ascorbate peroxidase (APX) activity was determined by monitoring the decrease in absorbance at 290 nm as reduced ascorbic acid (AsA) was oxidized (extinction coefficient of 2.8/mM/cm) (Yu et al., 2009). Phenylalanine ammonia-lyase (PAL) activity was determined spectrophotometrically, by measuring the amount of trans-cinnamic acid formed at 290 nm (extinction coefficient of 17.4/mM/cm) (Wang et al., 2011). The enzyme reaction mixture system that lacked L-phenylalanine was used as the control.

### RNA Extraction and Quantitative Real-Time PCR

Total RNA was extracted from the tomato tissues using an RNA extraction kit (Tiangen, Shanghai, China). Total RNA was quantified on a Nano Drop™ 1000 spectrophotometer before and after DNase I treatment, and its quality and integrity were checked by electrophoresis through agarose gels stained with ethidium bromide. The RNA was reverse-transcribed to generate cDNA using the Rever Tra Ace q-PCR RT kit (Toyobo, Tokyo, Japan) by following the manufacturer’s instruction. The tomato actin gene was used as an internal control. Gene-specific primers were designed according to cDNA sequences as described in Table 1. The qRT-PCR was performed with SYBR Green PCR Master Mix (Takara, Tokyo, Japan) in CFX connect RT-PCR detection system (Bio-Rad, Hercules, CA, United States). The PCR conditions consisted of denaturation at 95°C for 1 min followed by 40 cycles of denaturation at 95°C for 20 s, annealing at 60°C for 20 s, and extension at 72°C for 30 s. Each qRT-PCR was repeated three times. The 2^–ΔΔ*CT*^ method was followed for determining the relative expression of target genes.

**TABLE 1:** Sequences for primers used in the quantitative real time PCR (qRT-PCR) analysis.

Gene	Accession no.	Primer sequence (5′–3′)
PR1		F: 5′-GCT CAA AAC TCC CCT CAA-3′
		R: 5′-TGC TTC TCA TCA ACC CAC-3′
PR2		F: 5′-AAGTATATAGCTGTTGGTAATGAA-3′
		R: 5′-ATTCTCATCAAACATGGCGAA-3′
NP24		F: 5′-GCT CCG AGG GGA ACT AAG A-3′
		R: 5′-ACC AGG GCA AGT AAA TGT G-3′
Actin		F: 5′-CTAAGGCTAATCGTGAGAA-3′
		R: 5′-CGTAAATAGGAACCGTGT-3′

### Statistical Analysis

There were at least three independent replicates used for each determination, and average figures would be available. A statistical analysis of the bioassays was performed with the SPSS 16.0 statistical software package; all data were analyzed by one-way ANOVA, followed by Duncan’s multiple range tests. A *p*-value of < 0.05 indicates a significant difference, and data were presented as the mean ± standard deviation of three replicate samples.

## Results

### Control Effects of Melatonin and Selenium on Gray Mold Disease

**FIGURE 1:**
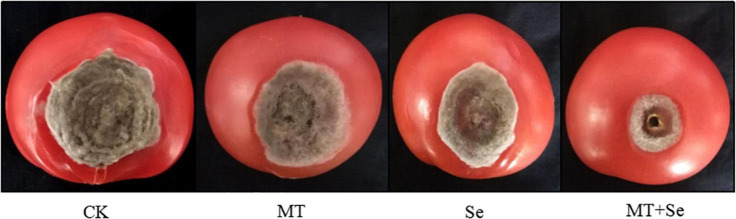
The inhibitory effect of melatonin (MT) and selenite (Se) treatments on the postharvest gray mold disease spots of tomato fruit. Tomato fruit was treated with tap water only (CK); 10 μmol/L MT; 20 mg/L Se from sodium Se; 10 μmol/L MT and 20 mg/L Se (MT + Se).

**FIGURE 2:**
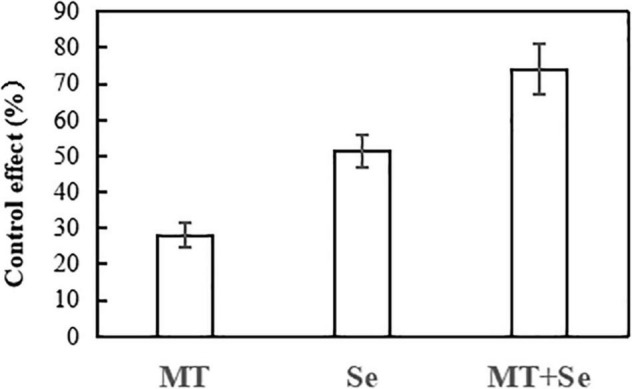
The control effect of MT and Se on the spot of postharvest gray mold of tomato fruits. Error bars indicate the standard error of three replicates. Different letters are significantly different according to the Duncan’s multiple range test (*p* < 0.05). 10 μmol/L melatonin (MT); 20 mg/L Se from sodium selenite (Se); 10 μmol/L MT, and 20 mg/L Se (MT + Se).

The Se treatment of 20 mg/L significantly inhibited the growth of gray mold *B. cinerea* in lesion areas in comparison with the control, but much effective inhibition was observed in the treatments of Se and MT interaction (Figure 1). The Se treatment significantly reduced the gray mold growth by 51%, while the MT treatment only reduced the effect of *B. cinerea* disease by 27%. However, the combination of both Se and MT (or the Se + MT treatment) resulted in the highest prevention effect of 74%. The fruit decay process was obviously suppressed by the Se + MT treatment 7 days after at 25°C (Figure 2).

### Effects of Melatonin and Selenium on the Growth of *Botrytis cinerea* Strain

**FIGURE 3:**
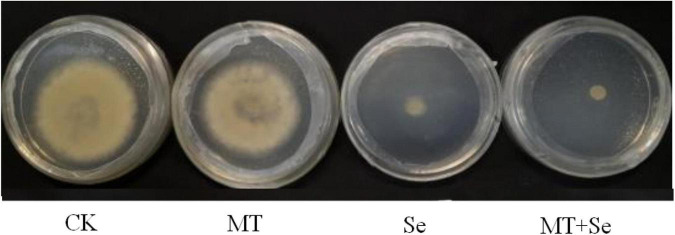
The inhibitory effect of MT and Se on the growth of postharvest pathogen *Botrytis cinerea*. *B. cinerea* was treated with sterile water only (CK); 10 μmol/L melatonin (MT); 20 mg/L Se from sodium selenite (Se); 20 mg/L Se and 10 μmol/L MT (Se + MT).

**FIGURE 4:**
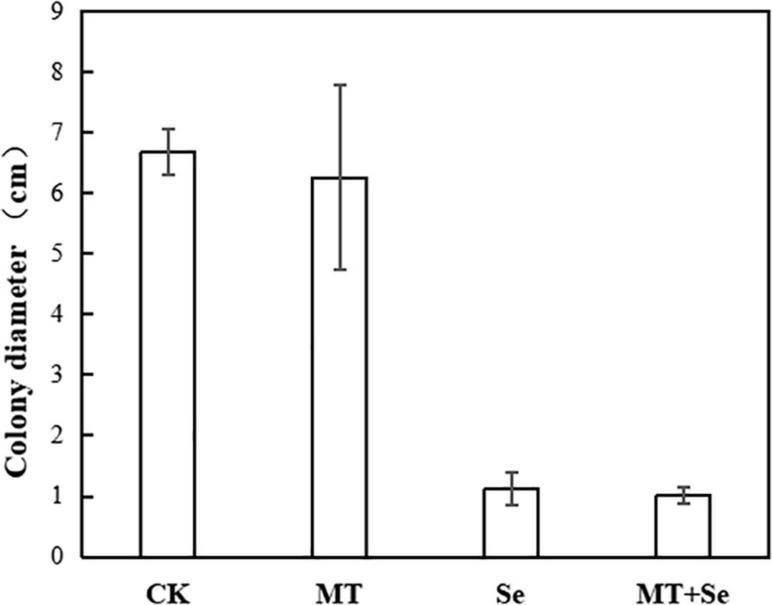
The inhibitory effect of MT and Se on the colony diameter (cm) of postharvest pathogen *B. cinerea*. Error bars indicate the standard error of three replicates. Different letters are significantly different according to the Duncan’s multiple range test (*p* < 0.05). Tomato fruit was treated with the sterile water only (CK); 10 μmol/L melatonin (MT); 20 mg/L Se from sodium selenite (Se); 10 μmol/L MT; and 20 mg/L Se (MT + Se).

The MT treatment did not show any significant effects on the colony diameter of *B. cinerea* strain compared with control, but the Se and the Se + MT treatments showed a significant decrease in colony diameter (Figure 3). It was not statistically significant for the inhibition of *B. cinerea* strain growth in PDA medium treated with only MT (Figure 4). Interestingly, although the MT treatment showed little effective inhibition on fruit decay after the pathogen inoculation, the Se + MT treatment significantly reduced the incidence and lesion diameter of gray mold on tomato fruits.

### Antioxidative Enzymes and Disease-Related Enzymes Activity

**FIGURE 5:**
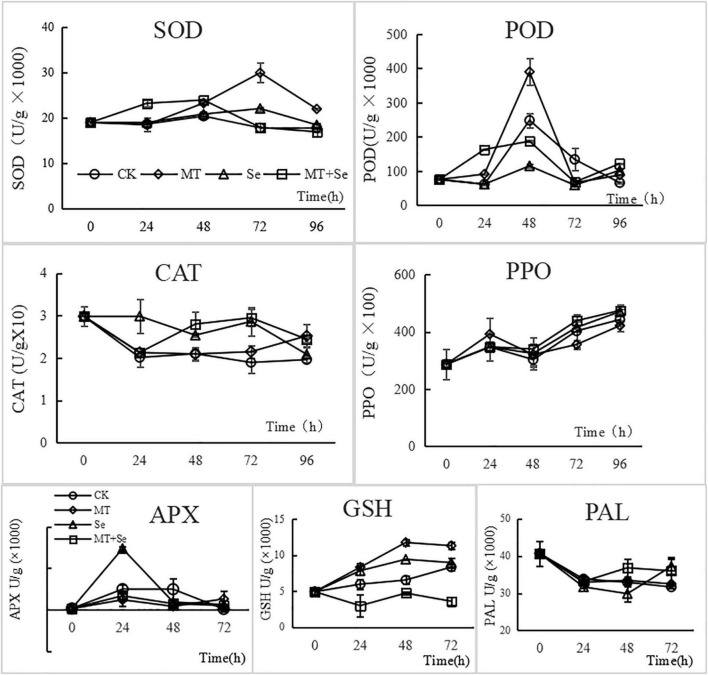
Effects of MT Se treatment on the activities of superoxide dismutase (SOD), peroxidase (POD), catalase (CAT), Polyphenol oxidase (PPO), ascorbate peroxidase (APX), glutathione (GSH), and phenylalanin ammonia-lyase (PAL) enzymes in tomato fruit. Error bars indicate the standard error of three replicates. Different letters are significantly different according to the Duncan’s multiple range test (*p* < 0.05). After fruit wound was added with 20 μl of the different treatment solutions: Se (20 mg/L), MT (10 μmol/L) and Se + MT (20 mg/L Se + 10 μmol/L MT), SOD, POD, CAT, PPO, APX, GSH, and PAL were determined at 0, 24, 48, 72, and 96 h, respectively.

Both MT and Se treatments significantly enhanced the activities of SOD after 48 h in tomato fruits, while the Se + MT increased the activities of SOD at 24 h. Although the effect of Se + MT was not as high as MT, it was earlier to increase the activities of SOD. A similar pattern of increase was found with POD except for inhibition of Se on POD (Figure 5), and both MT and Se could decrease the CAT activity in tomato fruits, but had little effect on PPO (Figure 5). The Se + MT treatment could increase the GSH content in tomatoes, but the Se + MT treatment led to the decrease of GSH content. Besides, the only Se treatment improved the activity of APX in tomato fruits, but there were no effects on PAL activities. The treatment effect on each enzyme selected was described below.

After challenge-inoculation with *B. cinerea*, the SOD activity of tomato fruits increased first and then decreased. The MT and Se treatments both enhanced the activity of SOD in tomato fruits, showing 68.03% by the MT treatment and 23.82% increase by the Se treatment, compared with the control, at 72 h after the inoculation (Figure 5). The SOD activity was significantly (*p* < 0.05) increased by 24.53% from the Se + MT treatment 24 h after, compared with the control. The MT treatment increased the activity of POD in tomato fruits, while the Se treatment significantly reduces the POD activity. However, the Se + MT treatment significantly increased the POD activity, compared with the control. The enzyme activity showed a trend of increasing first and decreasing later after the inoculation, and it reached the highest at 48 h. The SOD activity increased by 57.10% at 48 h compared with the control in the MT treatment, while the POD activity in tomato fruit was significantly increased by 47.6% at 24 h after inoculation in the Se + MT treatment. The control CAT activity decreased first and then remained stable during the first 24 h (Figure 5). However, the decrease in the POD activity in tomato fruits in the Se treatment and the Se + MT treatment was alleviated. The effect of MT was not statistically significant before 72 h (*p* > 0.05), but it significantly increased by 28.55% (*p* < 0.05) at 96 h. In general, the MT and Se treatments could slow down the decreasing process of the CAT activity after inoculation.

Neither the MT treatment nor the Se treatment showed significant impacts on the PPO activity of tomato fruits. After the treatments, the PPO enzyme activity increased during the first 24 h, decreased during the time period of 24–48 h, and then increased again (Figure 5). There was no significant difference in the PPO activity between the MT, the Se, and the Se + MT treatments after inoculation. The Se treatment increased the APX activity significantly (195.7%) from tomato fruits compared with the control at 24 h after inoculation (Figure 5). However, there was no significant difference in the PPO activity among the MT, the Se, and the Se + MT treatments after inoculation.

After inoculation, the GSH content in tomato fruits showed a trend of increasing and then stabilizing. Both the MT and the Se treatments increased the GSH content in fruits compared with the control, showing that the MT treatment was more effective than the Se treatment. Specifically, the content of GSH in the MT treatment increased by 35.49–79.59%, while the content of GSH in the Se treatment increased by 7.38–43.96% compared with the control (Figure 5). What interesting is that the Se + MT treatment did not increase the content of GSH but decreased the content by 27.76–56.86%. This suggested that the MT and the Se treatments had an antagonistic effect on the content of GSH in tomato fruits. The PAL activity of tomato fruits showed a trend of declining, and both the MT and the Se treatments had no significant effects on the PAL activity of fruit. The PAL activity in the Se + MT treatment was slightly higher than that of the control (11.84–13.69%, *p* > 0.05; Figure 5).

### The Quantitative Real-Time PCR Verification of the Target Gene Expression Levels

**FIGURE 6:**
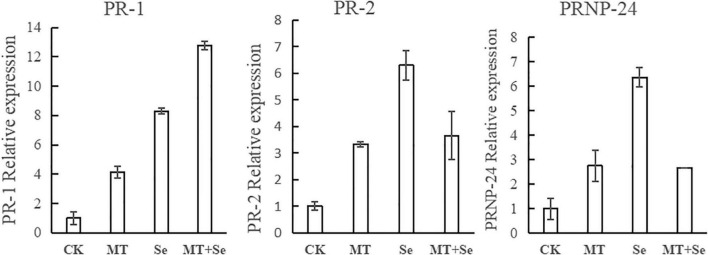
After 24 h of MT and Se treatments on fruit, its total RNA was extracted and transcript abundance of PR-1, PR-2, and PR-24 were determined by the quantitative RT-PCR. Error bars indicate the standard error of three replicates. Different letters are significantly different according to the Duncan’s multiple range test (*p* < 0.05).

While studying the expression of pathogenesis-related (PR) protein genes, different accumulation patterns of PR-protein mRNAs in Se-Mel-treated tomato fruits were observed. The expression of PR-1 in the tomato fruits from the MT and the Se treatments was about four and eight times as much as that of the control group, respectively, while the induction effect of the Se + MT treatment was significant, and PR-1 was more than 12 times higher than that of the control group (Figure 6). However, the Se treatment alone had the best effect on both of PR-2 and PR-24. The expression of PR-2 and PR-24 in the Se treatment was 6–7 times higher than that of the control, and the PR-2 and PR-24 in the MT and the Se + MT treatments was 2–4 times higher than that of the control (Figure 6). The effect of the Se treatment is better than those of the MT treatment and the Se + MT treatment.

## Discussion

### Effect of Trace Mineral Nutrient Selenium on Controlling Plant Diseases

Since environmental pollution and food safety as well as pesticide resistance caused by the application of traditional chemical pesticides to control the plant diseases, the demand of environmentally friendly ways to control plant diseases has become an important research topic (Datnoff et al., 2007). Among of them, mineral nutrition such as silicon (Si) has long been recognized as an important component of disease control practices, which impacts plant disease tolerance or resistance (Datnoff et al., 2007; Fones et al., 2010; Yao et al., 2012; Fones and Preston, 2013; Hörger et al., 2013; Kaur et al., 2016; Morkunas et al., 2018; Cabot et al., 2019; Jali et al., 2019; Liu et al., 2022). Solid research evidences demonstrate that the amounts of trace element nutrient Se in plants improve the plant growth and increase the tolerance against biotic and abiotic stress (Razak et al., 1991; Poschenrieder et al., 2006; Boyd, 2007; Freeman et al., 2007; Trumble and Sorensen, 2008; Pilon-Smits et al., 2009; Zhao and McGrath, 2009; Zhu et al., 2009; Winkel et al., 2011; Wu et al., 2015; Hossain et al., 2021) and appropriate Se can significantly enhance the antioxidant enzyme activities in plants and reduce the activities of reactive oxygen species (ROS) and also the levels of lipid peroxidation, which could enhance the antioxidant enzyme activities in plants (Feng et al., 2013; Lanza et al., 2021). Selenium could control the *Sclerotinia* stem rot disease of plants by regulating the soil microorganism community (Liu et al., 2019a). Se significantly inhibits the spore germination of fungal pathogen and effectively controlled gray mold growth in harvested tomato fruit. The Se action on the *B. cinerea* growth was due to conidia plasma membrane damage and cytoplasmic materials loss (Wu et al., 2014, 2016; Youssef and Roberto, 2014; Zhu et al., 2016, 2017). In the present study, Se inhibited the plant pathogenic fungi and could improve the enzyme activity of defense system, such as SOD, CAT, and APX, as well as the expression of disease-related protein genes (PR-1, PR-2, and PR-24). Our results showed similar results that Se is effective in inhibiting the mycelial growth of *S. sclerotiorum* strain, decreasing SOD, and CAT activities (Shi et al., 2011; Yaganza et al., 2014; Xu et al., 2020).

### Controlling Plant Diseases With Melatonin

Melatonin is involved in various responses in plants to biotic or abiotic stress (Paredes et al., 2009; Tan et al., 2012; Arnao and Hernandez-Ruiz, 2014, 2015; Reiter et al., 2015; Nawaz et al., 2016; Shi et al., 2016; Wang et al., 2018; Li et al., 2019; Zhao et al., 2021). The previous studies have shown that MT can help in controlling the plant diseases (Yin et al., 2013; Shi et al., 2015; Zhao et al., 2015; Mandal et al., 2018). Leaves treated with MT exhibited the delayed senescence, which may be due to its roles in scavenging free radical or inhibiting the senescence-associated genes (Sun et al., 2015; Wang et al., 2020; Ze et al., 2021). The data here indicated that MT had no significant effects on the postharvest gray mold of tomato fruit, but could induce the enzyme activity of fruit defense system and activate the expression of proteins related to disease course. Our findings are similar with the results of apple trees with MT treatments enabled plants to maintain intracellular H_2_O_2_ concentrations at the steady-state levels and enhance the activities of defense-related enzymes, possibly improve disease resistance (Yin et al., 2013; Sun et al., 2015; Xu et al., 2019; Wang et al., 2020; Ze et al., 2021). Since MT acts upstream of the synthesis of salicylic acid (SA), the MT levels in the knockout *Arabidopsis* may have been responsible for the SA levels, and related to plant resistance (Zhao et al., 2015; Li et al., 2019; Lin et al., 2019; Liu et al., 2019b). MT enhances the pathogen resistance by inducing the expression of a number of plant defense-related genes (Shi et al., 2015, 2016; Li et al., 2019; Liu et al., 2019b; Xu et al., 2019; Zhao et al., 2021). Our results strongly supported the idea that MT may be a defense signaling molecule in plants against pathogens.

### Synergistic Interactive Effects of Selenium and Melatonin on Abiotic and Biotic Plant Stresses

Selenium plays a role in the prevention of oxidative damage and melatonin has an antioxidant effect which protects against more free radical species. Recently, some studies have indicated that the protective and synergistic scavenger effects of Se with vitamin E and MT on toxic-induced oxidative damage in animals, is also called Se-Mel (Kara et al., 2008; Ahmad et al., 2011). In addition, Se could induce the synthesis of MT in plants, and MT participates in Se-enhanced stress tolerance in plant (Li et al., 2016; Sun et al., 2021; Yang et al., 2021; Farooq et al., 2022). In the present study, the results showed that the effect of treatment with MT and Se was better than that of Se or MT alone on the disease control and prevention. Furthermore, the Se and MT treatment could induce the disease resistance of tomato by improving the enzyme activity of immune defense mechanism system and up-regulating the gene expression of pathogenesis-related proteins (PR-1, PR-2, and PR-24). Also, endogenous MT and Se accumulation is warranted to be further investigated for discovering possible interactions between them. Also, the effects of MT and Se biosynthesis genes expression are warranted to be deeply studied for a better understanding of employed mechanisms by MT and Se treatment for conferring gray mold decay resistance in tomato fruits. In summary, the results indicate that the inhibition effect of MT and Se on gray mold disease of tomato fruits causes by *B. cinerea* and reported that combined MT and Se treatment significantly attenuated gray mold decay in tomato fruits during 7 days of storage at 25°C. Exogenous MT and Se treatment promoted the ROS scavenging enzymes activity and triggered PRs genes expression.

## Conclusion

In this study, we evaluated the impact of MT and Se on gray mold disease of tomato fruit caused by *B. cinerea* and reported that combined melatonin and selenium treatment significantly attenuated gray mold decay in tomato fruits during 7 days of storage at 25°C. Exogenous MT and Se treatment promoted the ROS scavenging enzymes activity and triggered PRs genes expression. The tomato fruits treated with exogenous MT or Se showed improved resistance to gray mold induced by *B. cinerea*, and the treatment of Se and MT could induce the disease resistance of tomato fruits by improving the antioxidative enzymes activity in harvest fruit and the expression of PR-1, PR-2, and PR-24 genes. The combined MT and Se interaction had the most significant effect on the PR-1 gene expression. These results suggested that MT and Se interaction might be promising as an environmentally friendly technique to fight against postharvest decay of fruit.

## Data Availability Statement

The original contributions presented in the study are included in the article/supplementary material, further inquiries can be directed to the corresponding authors.

## Author Contributions

HZ and ML designed the experiments and wrote the main manuscript text. HZ and JM prepared all figures and tables. GB, Z-QL, and RR revised the manuscript. ZW, XY, LY, and RZ provided advices and assistance to the submission of the final manuscript. All authors reviewed the manuscript and approved the submitted version.

## Conflict of Interest

The authors declare that the research was conducted in the absence of any commercial or financial relationships that could be construed as a potential conflict of interest.

## Publisher’s Note

All claims expressed in this article are solely those of the authors and do not necessarily represent those of their affiliated organizations, or those of the publisher, the editors and the reviewers. Any product that may be evaluated in this article, or claim that may be made by its manufacturer, is not guaranteed or endorsed by the publisher.
